# 165. Changing the Culture of Obtaining Urine Cultures; Diagnostic Stewardship Intervention Leading to Sustained Reduction in Inpatient CAUTI Rates 

**DOI:** 10.1093/ofid/ofad500.238

**Published:** 2023-11-27

**Authors:** Cristina J Torres, Elizabeth Lyden, Trevor C Van Schooneveld, Mark E Rupp

**Affiliations:** University of Nebraska Medical Center, Papillion, Nebraska; University of Nebraska Medical Center, Papillion, Nebraska; University of Nebraska Medical Center, Papillion, Nebraska; University of Nebraska Medical Center, Papillion, Nebraska

## Abstract

**Background:**

Asymptomatic bacteriuria (ASB) is common in patients with urinary catheters and unneeded urine cultures (UC) can lead to misidentification of CAUTI. We evaluated the long-term effectiveness of restricting UC utilization on the incidence of inpatient CAUTI and if the COVID pandemic influenced CAUTI rates.

**Methods:**

A UTI evaluation panel was implemented at our academic medical center in April 2015 requiring clinician documentation of UTI symptoms or criteria supporting UC in the absence of symptoms or pyuria. UC was performed reflexively if the following were present: documented symptoms, pyuria ( > 10WBC), and no contamination ( > 100 squamous cells) (Figure 1). UC in at risk patients without symptoms or pyuria were performed. CAUTI data was collected using NHSN methodology with 2014 CAUTI rates recalculated using 2015 definitions. Three periods were compared: Pre-implementation (1/2014-3/2015), post-implementation (4/2015-3/2020) and COVID (4/2020-6/2022). NHSN SIR and SUR were compared where available (4/2015-3/2020 vs. 4/2020-6/2022). Poisson regression was used to model rate of infection per month. A generalizing estimating equation was used for interrupting time series model to compare rates of infection.

**Figure 1:**
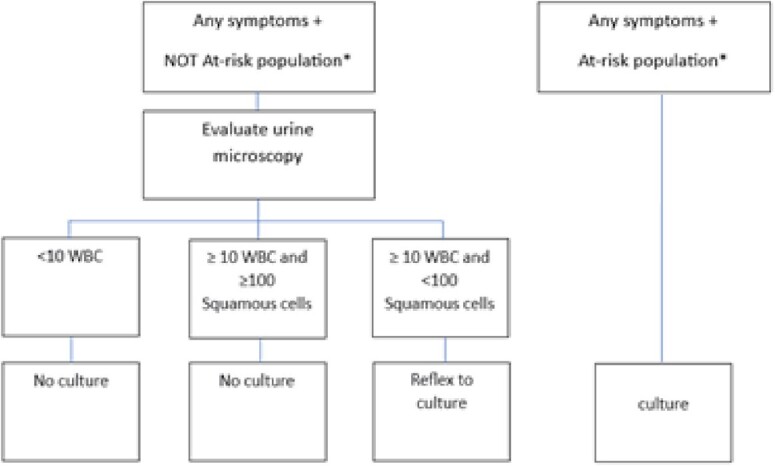
Urinalysis algorithm *At-risk population: neutropenia, kidney/pancreas transplant, pregnancy, impending urologic surgery, age <3 years

**Results:**

Urine culture rates decreased in both post and COVID periods and catheter days decreased in the post but not COVID period (Table 1). CAUTI rates per 1000 CD decreased 40% between pre- and post-intervention (p=0.0001) and were unchanged during COVID, (p=0.61) although the slope of change did alter from decreasing to increasing (Figure 2). Quarterly SURs decreased during COVID while the institutional SIR increased non-significantly.

**Table 1:**
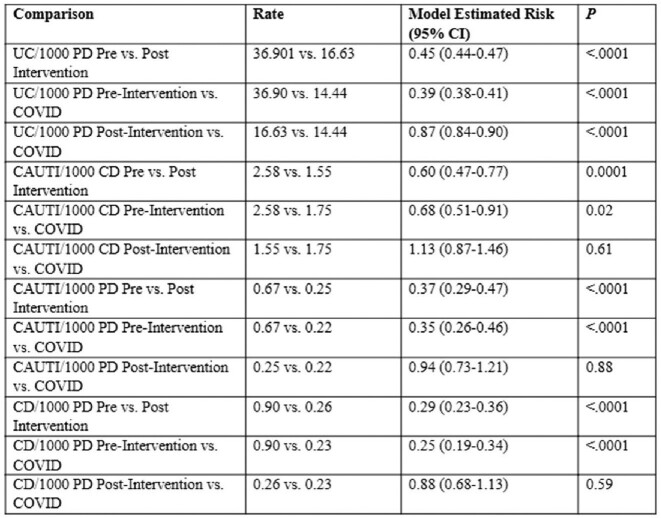
Comparison of Change in Urine Culture CAUTI and Catheter Days Between Periods (Pre=1/2014-3/2015; Post=4/2015-3/2020; COVID=4/2020-6/2022). UC=Urine cultures; PD=Patient days; CAUTI=Catheter-associated UTI; CD=Catheter days

**Table 2:**
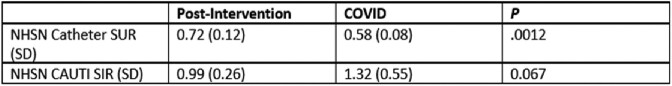
Median Quarterly Urinary Catheter SUR and CAUTI SIR Post-Intervention (Q1/2016-Q1/2020) Compared to COVID (Q2/2020-Q2/2022) SUR=Standardized Utilization Ration; SIR=Standardized Infection Ratio

Figure 2:

**Figure:**
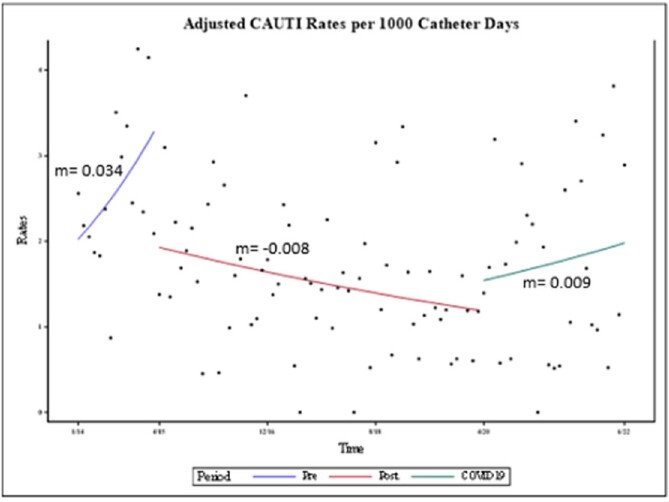

**Conclusion:**

Our intervention decreased urine cultures and CAUTI. There was a non-statistically significant increase in CAUTI during the COVID pandemic with the direction of rate of change going from negative to positive. This may have been due to diversion of resources from infection prevention programs. Catheter use decreased throughout; this may have impacted the metrics which used CD as a denominator (CAUTI, NHSN SIR) as both showed non-significant increases during COVID.

**Disclosures:**

**Trevor C. Van Schooneveld, MD, FSHEA, FACP**, AN2 Therapeutics: Grant/Research Support|Biomeriuex: Advisor/Consultant|Biomeriuex: Grant/Research Support|Insmed: Grant/Research Support|Thermo-Fischer: Honoraria **Mark E. Rupp, MD**, 3M: Advisor/Consultant|Citius: Advisor/Consultant|contrafect: Grant/Research Support|Teleflex: Advisor/Consultant

